# Drone exposure to the systemic insecticide Fipronil indirectly impairs queen reproductive potential

**DOI:** 10.1038/srep31904

**Published:** 2016-08-23

**Authors:** Guillaume Kairo, Bertille Provost, Sylvie Tchamitchian, Faten Ben Abdelkader, Marc Bonnet, Marianne Cousin, Jacques Sénéchal, Pauline Benet, André Kretzschmar, Luc P. Belzunces, Jean-Luc Brunet

**Affiliations:** 1 INRA, UR 406 Abeilles & Environnement, Toxicologie Environnementale, Avignon, 84914, France; 2 SUPAGRO, Laboratoire de Pathologies de l’Abeille, Montpellier, 34090, France; 3 INAT, Laboratoire de Zoologie et d’Apiculture, Tunis, 1082, Tunisie; 4 INRA, UR 546 Biostatistiques & Processus Spatiaux, Avignon, 84914, France

## Abstract

A species that requires sexual reproduction but cannot reproduce is doomed to extinction. The important increasing loss of species emphasizes the ecological significance of elucidating the effects of environmental stressors, such as pesticides, on reproduction. Despite its special reproductive behavior, the honey bee was selected as a relevant and integrative environmental model because of its constant and diverse exposure to many stressors due to foraging activity. The widely used insecticide Fipronil, the use of which is controversial because of its adverse effects on honey bees, was chosen to expose captive drones in hives via syrup contaminated at 0.1 μg/L and gathered by foragers. Such environmental exposure led to decreased spermatozoa concentration and sperm viability coupled with an increased sperm metabolic rate, resulting in drone fertility impairment. Subsequently, unexposed queens inseminated with such sperm exhibited fewer spermatozoa with lower viability in their spermatheca, leaving no doubt about the detrimental consequences for the reproductive potential of queens, which are key for colony sustainability. These findings suggest that pesticides could contribute to declining honey bee populations through fertility impairment, as exemplified by Fipronil. More broadly, reproductive disorders should be taken into consideration when investigating the decline of other species.

Over recent decades, Earth has been experiencing a major crisis due to an alarming rate of species loss, suggesting a sixth mass extinction^1^. In this context, more than 25% of studied plant and animal species are threatened^2^. Among pollinating insect species, data available from several studied bee species, such as bumble bees and honey bees, have shown the existence of some cases not only of population decline but also of general or relatively localized extinction^3^. The anthropogenic loss of species is the result of major threats, such as the exploitation of resources, the destruction and fragmentation of habitats, the introduction of invasive species, and the spread of diseases and industrial or agrochemical pollutants^1^,^4^. Two stressors in particular, pesticides and diseases, are known to disrupt reproductive function, which is essential for species survival. Some of these environmental stressors can directly disrupt fertility in humans, as observed during the last century^5^, either directly or through the endocrine system, which plays a central role in reproductive functions^6^. Such reproductive disorders have been reported in many species other than humans^7^, and especially in some insects, manifested by mating behavior and fertility impairments^8^,^9^,^10^,^11^,^12^, suggesting the potential for similar fertility effects in the honey bee (*Apis mellifera* L.).

Honey bees are social insects of great economic and ecological interest, and declining populations have been reported over the last three decades^13^,^14^. Among the suspected causes of this decline^14^, queen failure appears to be particularly significant^15^,^16^,^17^. The symptoms of this phenomenon include the following: decreases in or an absence of egg laying; the appearance of a lacunar brood, i.e., an abnormal brood surface exhibiting scattered empty cells; the excessive laying of unfertilized eggs, resulting in drone births; and early supersedure or queenless colonies^14^,^18^. These symptoms may be due to queen exposure to pesticides^19^,^20^,^21^,^22^ and biological agents, such as the ectodermic parasite *Varroa destructor*, the microsporidium *Nosema ceranae*, or viruses^18^,^23^,^24^. A shortage of available healthy drones (i.e., male honey bees) with high-quality semen may also cause queens to become poorly mated, thus diminishing queen quality^18^,^25^,^26^. Although queens have been studied more often than drones, few reports have characterized the impacts of environmental stressors on drone survival and fertility. These studies are mainly focused on the effects of parasites and veterinary treatments^26^,^27^,^28^.

However, no studies have established a direct relationship between insecticides, drone fertility, and the final impact on the queen. Thus, we assessed whether reduced queen quality could also be the result of disrupted drone fertility induced by sublethal exposures to insecticides. Our attention was focused on Fipronil, a pesticide widely used for its systemic properties^29^ in numerous varieties of agricultural and veterinary applications. Because Fipronil diffuses into plant tissues, it contaminates nectar and pollen, which are food sources for the honey bee^29^. In addition, honey bees in hives could be exposed to Fipronil when it is used to control the small hive beetle *Aethina tumida*^30^. This neurotoxic phenylpyrazole insecticide is known to induce lethal and sublethal effects at the cellular^31^,^32^, behavioral^33^,^34^,^35^,^36^ and colonial level^36^ in honey bees as well as in non-target invertebrates^37^. Interestingly, Fipronil also alters the reproductive functions of some terrestrial and aquatic vertebrates and invertebrates^38^,^39^,^40^,^41^,^42^. To study the impact on fertility, drones, which depend on workers for their food supply, were chronically exposed throughout their sexual maturation to a contaminated food source gathered by foragers. This food was a syrup solution contaminated with Fipronil at 0.1 μg.L^−1^ (0.087 μg.kg^−1^; density, d = 1.1488), which is an environmentally relevant concentration^29^. The impacts of this exposure on foraging activity and drone life cycle traits and sperm quality were monitored. The impacts on queen fertility were also investigated by analyzing the spermathecal content of queens instrumentally inseminated with sperm from exposed or unexposed drones. The results are discussed in terms of potential impacts on queen performance at the colony level.

## Results and Discussion

### Fipronil affects male fertility

**Figure 1:**
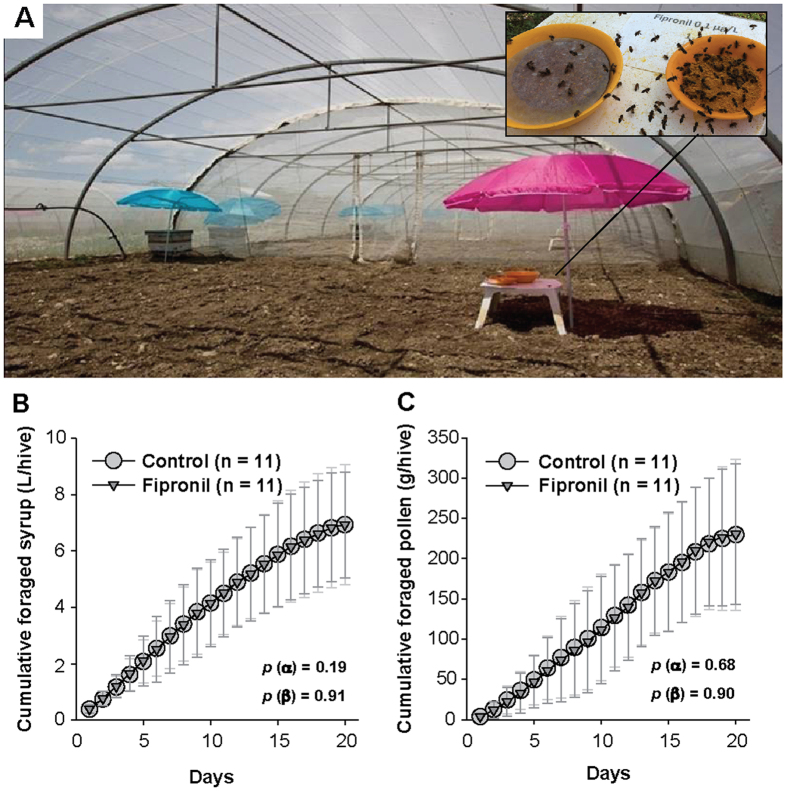
Foraging behavior in colonies. For 20 days, from drone emergence to sexual maturity, colonies were supplied daily with sugar syrup (50% w/v) *ad libitum* using a feeder, for harvesting by foragers from 8:30 a.m. to 11:30 a.m.; the syrup was replaced with crushed pollen and water for the remainder of the day. While the hives were fed during the exposure period, foraging behavior was monitored in 44 colonies from 22 tunnel compartments. (**A**) Illustration of the experimental platform; (**B**) cumulative foraged syrup; and (**C**) cumulative foraged pollen. The data represent the mean± the standard deviation of the daily cumulative foraged quantity observed in each compartment (1 feeder for 2 hives). For each treatment, the data correspond to the set of values from 4 experiments conducted between 2012 and 2014 (n = 11). Statistical analyses of the growth rates of cumulative foraging (α) and maximum cumulative quantities (β) were performed using a generalized linear mixed model with a random effect on the different experiments.

The apparent effects of sublethal exposure to Fipronil were investigated under semi-field conditions (Fig. 1A). No significant effects were observed on the foraging behavior of workers (Fig. 1B,C) or on drone survival (Table 1). These results are not surprising because behavioral effects of Fipronil are observed in worker bees only at concentrations ≥1 μg.kg^−1 ^33,34,36. However, no information about the effects of Fipronil on drones was available. In this context, drones were exposed to Fipronil via food brought back to the hive by foragers during the period from emergence to drone sexual maturity. However, drones directly consuming the syrup with Fipronil at 0.1 μg/L upon the return of foragers cannot be excluded. In addition, drones could also have consumed contaminated food stored in the hive with concentrations of Fipronil different from that of the syrup. During hive storage and while the honey matures, Fipronil could be concentrated, partly transferred to bees or metabolized by bees or by enzymes excreted in honey^43^, and metabolites could appear. Thus, drone exposure is not directly controlled during this period. Hence, to assess the Fipronil exposure, the concentration of Fipronil and its metabolites in honey stored in hives after 20 days of foraging was determined using GC–MS/MS chemical analysis. However, the values of Fipronil and metabolite concentrations were lower than the limit of detection (LOD = 0.2 μg/kg of honey). Residues in males were not analyzed because we had conducted a preliminary assessment of the exposure. This latter assessment was based on the sugar consumption of drones (21–90 mg/drone/day^44^), the sugar concentration in the food (50% w/v), the exposure period (20 days) and the Fipronil concentration in the syrup (0.1 μg/L). Thus, in the worst case scenario (i.e., the highest food consumption, absence of elimination and complete bioaccumulation of the doses ingested), the cumulative dose of Fipronil ingested per drone during the exposure period was estimated at 360 pg/drone. Considering that drones weigh approximately 196–225 mg^45^, this cumulative amount of Fipronil corresponded to 1.84 μg of Fipronil per kg of drone for a drone weight of 196 mg; this amount was less than the LOD of the analytical method (3 μg/kg of bees). Despite this low exposure level, Fipronil affecting the development of drones could not be excluded. However, no effects on drone sexual maturity rate or semen volume were observed (Table 1).

**Figure 2:**
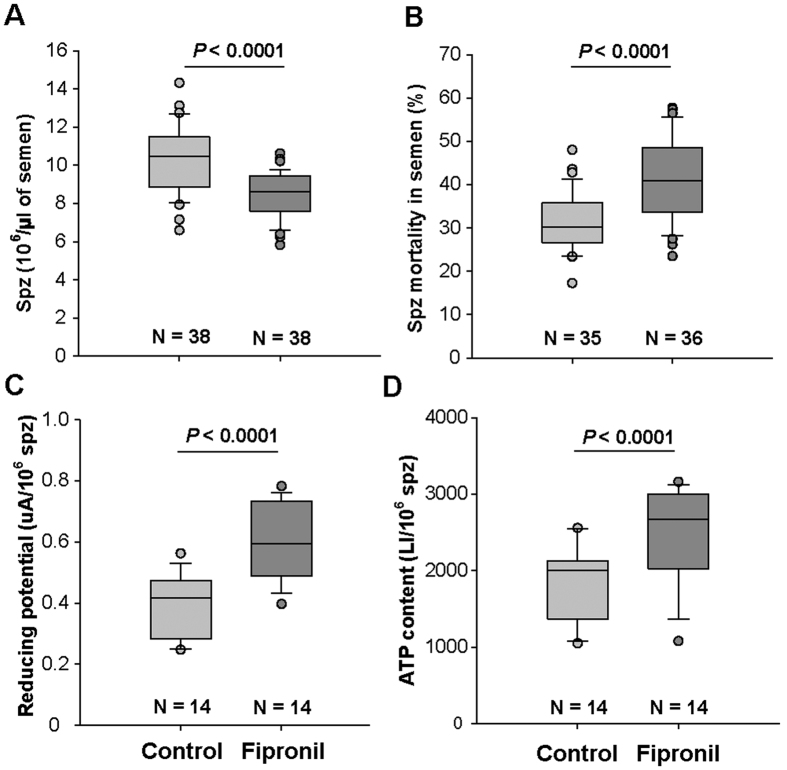
Effects of chronic exposure to Fipronil on drone fertility. The day after collection, the semen samples were analyzed to assess the effects of Fipronil exposure on drone fertility. (**A**) Total spermatozoa (spz) concentration in semen, including live and dead spermatozoa. (**B**) Mortality rate of spz expressed as a percentage (%). (**C**) Reducing potential of semen in absorbance units (AU λ = 570 nm), corresponding to the rate of reduced resazurin per million spz. (**D**) Rate of adenosine triphosphate (ATP) in spz, expressed as the luminescence intensity (LI) per million spz. For each parameter, the data correspond to the values obtained in experiments conducted between 2012 and 2014. The reducing potential and ATP content assays were only performed during the three first experiments; “n” indicates the number of samples for each treatment and parameter. Statistical analyses were performed using a generalized linear mixed model with a random effect on the different experiments.

Because no apparent effects were observed at the individual level, the quality of semen was studied in greater detail by analyzing sperm count and other physiological parameters. Drones achieve spermatogenesis at the larval stage. Thus, number of spermatozoa at emergence is determined early. Therefore, as exposure to Fipronil started at emergence, it could not affect the production of spermatozoa and could only affect seminal fluid production, which occurs during drone’s sexual maturation at approximately 15–20 days after emergence. During this period, spermatozoa mature in the seminal vesicles with the head anchored in the secretory epithelium and are released in the seminal gland at sexual maturity^46^. In the present study, the semen volume produced per drone was not affected by exposure to Fipronil, which also suggested that Fipronil has no apparent effect on semen quality (Table 1). Nevertheless, exposure to Fipronil induced a decrease in total spermatozoa concentration (median of 8.59 × 10^6^ spermatozoa vs. 10.47 × 10^6^ spermatozoa in controls, *P* < 0.0001, Fig. 2A) and an increased spermatozoa mortality rate (median of 40.91% vs. 30.32% in controls, *P* < 0.0001, Fig. 2B) in mature drones. Two hypotheses linking the decrease in spermatozoa concentration in semen to Fipronil exposure can be proposed: (i) a portion of the spermatozoa (dead or alive) remained bound to the seminal vesicle membrane and were not available in the ejaculated semen; and/or (ii) spermatozoa that died during the maturation process may also be lysed prior to ejaculation.

At the cellular level, reducing potential corresponds to the ability of cells to metabolically reduce compounds or to passively reduce oxidants resulting from metabolic activity or ionizing rays. Reducing potential was increased by exposure to Fipronil (median of 0.59 absorbance units (AU) vs. 0.42 AU in controls, *P* < 0.0001, Fig. 2C). The cellular ATP content produced by mitochondria was also increased (median of 2002.59 luminescence intensity (LI) vs. 2676.78 LI in controls, *P* < 0.0001, Fig. 2D). In spermatozoa, ATP corresponds to a supply of energy available not only for metabolic activity but also for motility. Similar metabolic modulations have been observed in other cell types as well as mitochondria^31^,^47^,^48^,^49^; in addition, depending on the mode of exposure, these modulations can lead to cell death^48^,^49^,^50^. We assume that these effects on the mortality and concentration of spermatozoa are due to physiological disruptions because an increase in energetic metabolism could lead to oxidative injuries by generating reactive oxygen species, as previously confirmed for Fipronil^38^,^49^,^50^. Although spermatozoa exhibit antioxidant defenses^51^, an overload of these defenses can lead to cell death, explaining the high mortality rate of spermatozoa in drones exposed to Fipronil. Moreover, the higher levels of ATP observed in the semen of exposed drones could be due not only to increased metabolism but also to decreased ATP consumption, resulting in lower sperm motility. This reduced ATP consumption could adversely impact the fertility of the queen because motility is involved in the competition between spermatozoa during the process of migrating to reach the spermatheca^52^ first and subsequently fertilize the eggs^53^. These results strongly suggest that the reproductive potential of drones could be affected by chronic exposure to Fipronil via food. These detrimental effects of Fipronil on drone sperm quality also suggest a potential impact on queen fertilization.

### Incidence on the queen

**Figure 3:**
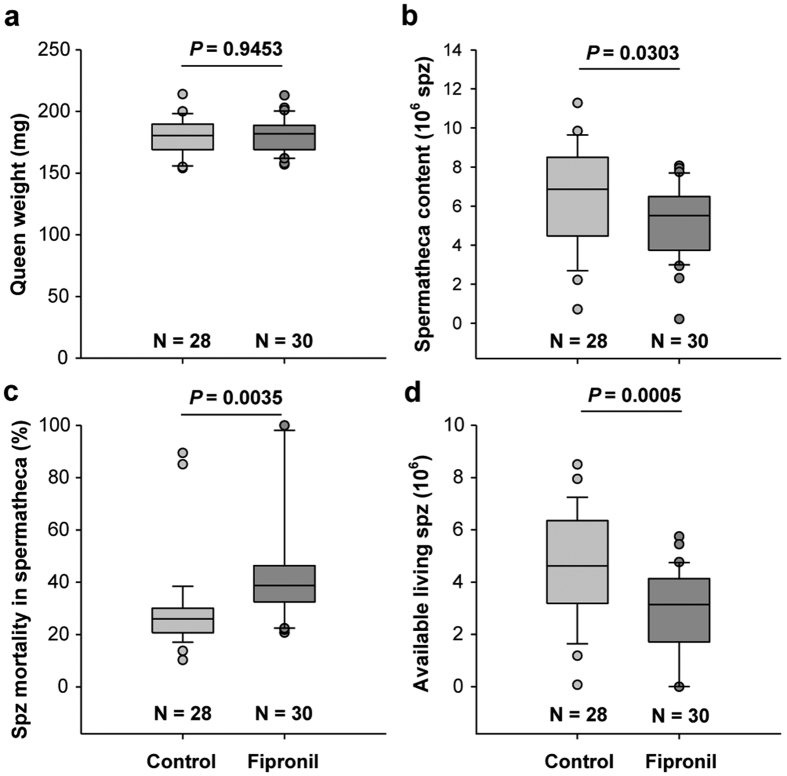
Consequences of drone exposure on queens. Following the exposure of drones in the experiment conducted in 2014, a portion of the collected semen was used to instrumentally inseminate 2 groups of 40 queens each. Two weeks later, the surviving queens in the control (n = 28) and Fipronil groups (n = 30) were weighed and dissected, and the spermathecae were analyzed to determine (**a**) queen weight, (**b**) the number of spermatozoa (spz) stored in the spermatheca, (**c**) the mortality rate of spz expressed as a percentage (%) of stored spz, and (**d**) live spz available to fertilize eggs, deducted from the two previous parameters. A t-test was applied to statistically analyzed queen weight (**a**). For parameters (**b**–**d**), statistical analyses were performed using a generalized linear mixed model with a random effect on the hive from which the sperm capillaries originated.

The effects of drone exposure to Fipronil on the queen were investigated by analyzing the weight and spermathecal content of sister queens 14 days after they were instrumentally inseminated with a fixed volume of semen (8 μL)^54^. No significant differences were observed in the weights of queens inseminated with the semen of drones exposed or not exposed to Fipronil (Fig. 3a). Conversely, queens inseminated with semen from exposed drones stored fewer spermatozoa (median: 5.52 × 10^6^ vs. 6.86 × 10^6^ in the controls, *P* = 0.0303, Fig. 3b) and exhibited a greater proportion of dead spermatozoa (median: 38.72% vs. 25.96% in the controls, *P* = 0.0035, Fig. 3c). Consequently, a 30% decrease in live spermatozoa recovered in spermatheca (median values: 4.62 × 10^6^ in treatment vs. 3.14 × 10^6^ spermatozoa in the control, *P* = 0.0005, Fig. 3d) was observed, reflecting a decrease in egg fertilization potential. The volume of the spermatheca is known to be correlated with the queen’s body weight^19^, which was identical in the two queen groups. Thus, the differences in spermatheca content in the two queen groups are attributable to differences in semen properties instead of queen physiology. This unprecedented result demonstrates that the reproductive potential of the queen can be indirectly affected by a chemical stressor by altering the semen of exposed drones.

**Figure 4:**
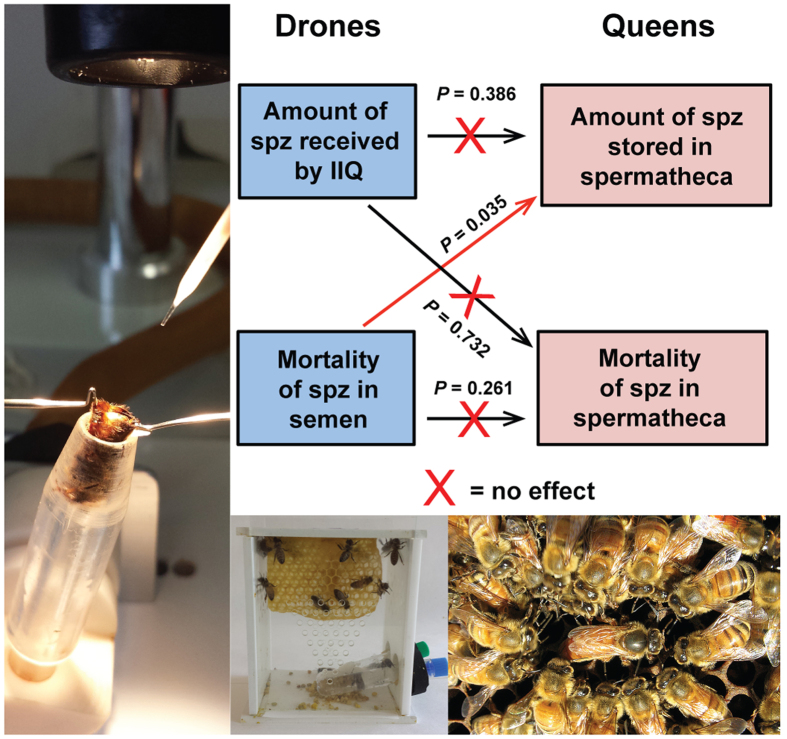
Relationships among drone semen characteristics and spermathecal content. For all of the instrumentally inseminated queens (IIQ) (n = 58), the quantity and mortality of injected spermatozoa (spz) were compared with those of spz stored in the spermatheca. Arrows correspond to the effect of one factor on another. The number of spz received by the queen was calculated based on the concentration and volume of injected spz (8 μL). Statistical analyses were performed using a generalized linear mixed model with a random effect on the hive from which the sperm capillaries originated. linear mixed model with a random effect on the different experiments.

These results suggest effects on sperm transfer mechanisms, which are somewhat complex in the honey bee. The similarity of the characteristics of the spermatheca content (numbers of total and live spermatozoa) to the injected semen suggests a passive transfer of spermatozoa from the oviduct to the spermatheca. After mating, the oviducts of queens receive an excess of approximately 90 × 10^6^ spermatozoa from several drones, and only a small portion (4 × 10^6^ to 7 × 10^6^) reaches the spermatheca by active migration or passive transport^52^,^55^. This suggests that a slight variation in the initial quantity of spermatozoa received by the queen does not influence the number of spermatozoa stored in the spermatheca. This assumption is consistent with the absence of a correlation between the initial number of spermatozoa in the semen used to inseminate the queens and the quantity of spermatozoa recovered in the spermatheca (*P* = 0.386, Fig. 4). Because the quality of semen in the spermatheca is superior to that in the oviduct^56^ and because dead spermatozoa do not migrate to the spermatheca^52^,^57^, the migration process may be considered as a mechanism of selecting the best spermatozoa for storage to compensate for lower sperm quality and to maintain the sustainability of the species. Our data indicate that this protective mechanism may be circumvented when fertilization is performed with altered semen from drones exposed to environmental pollutants, such as Fipronil, resulting in decreased semen numbers and mortality rate in the spermatheca. Considering all modalities together, only the number (*P* < 0.035, Fig. 4) but not the mortality rate (*P* = 0.261, Fig. 4) of spermatozoa stored in the spermatheca was affected by the number of dead spermatozoa in the semen during the migratory process. Considering the modalities separately, the discrepancy in the observed spermatozoa number and mortality in the spermatheca between the control and Fipronil modalities could have resulted from delayed effects of the drone exposure. These delayed effects could be consequences, at least in part, of the metabolic disruptions previously observed in the sperm, i.e., the increased reducing potential and ATP content. Thus, we hypothesize that the reduced quantity of spermatozoa recovered from the spermatheca of queens inseminated with semen from exposed drones could be also explained, independently of the phenomenon described above, by putative negative effects of such metabolic disruptions on the ability of spermatozoa to migrate. Similarly, we hypothesize that the higher mortality rate of spermatozoa observed in the spermatheca could be explained either by an impairment in the adaptation of spermatozoa having reached the spermatheca to the spermathecal fluid, which is necessary for long-term storage^58^, or by an increase of the mortality dynamic of the stored spermatozoa, which is a phenomenon that occurs naturally in the spermatheca over the time^59^.

These results demonstrate that the exposure of honey bee drones to pesticides such as Fipronil may have negative effects on the fertility of unexposed queens. Furthermore, the situation may be worse in reality, considering that additive effects may occur if queens are also exposed. Our findings raise questions about the fate of queens fertilized by drones exposed to pesticides and the sustainability of their colonies.

### Potential effects on queen offspring

Honey bee colonies exhibit high developmental potential due to the high activity of the queen, which can lay up to 200,000 eggs per year^45^. This high developmental potential is sustained by three main properties of the semen that is stored in the spermatheca of the queen and is linked to the polyandry practices of the queen. (i) The stored semen exhibits genetic diversity due to mating of the queen with as many as 20 drones^55^,^60^. This genetic diversity confers an evolutionary advantage that results in enhanced colony fitness via improved adaptation to environmental stressors^61^,^62^. (ii) The spermatheca contains a large number of spermatozoa^20^,^54^,^59^,^60^, and (iii) the spermatozoa exhibit a high viability rate^20^,^56^,^57^,^59^,^60^. The latter two properties, which were investigated in this study, are of high importance because they are integral parts of the important egg-laying activity that occurs for several years. Thus, the optimization of worker production relative to the requirements of the colony and the process of swarming to establish a new colony^45^ could be compromised. Because we observed that Fipronil elicits a decrease in both spermatozoa abundance and viability in the queen, Fipronil may have adverse and indirect impacts on queen performance, leading to a decrease in lifespan of the queen and/or in offspring abundance due to altered fertility of the queen.

Queen failure, or supersedure, is a main long-term effect of decreased spermathecal content quality. It is likely that a queen with a smaller number of live spermatozoa does not have the ability to lay fertilized eggs for as long as a queen with a better stock. In addition, a poor spermathecal content could quantitatively and qualitatively modulate the queen’s pheromone profile, which could become less attractive for workers, consequently impairing social interactions and colony cohesion^63^. These processes contribute to anticipated supersedure followed by a critical period during which there is not only an absence of brood production but also a risk that the new queen was killed in nuptial flight or poorly mated, with putative detrimental effects on colony sustainability.

The spermathecal content is also essential for the fertilization of eggs, a process that is poorly understood. It is not known whether queens release exclusively live spermatozoa or a random mixture of live and dead spermatozoa to fertilize eggs. If the queen releases a random mixture of live and dead spermatozoa, the higher abundance of dead spermatozoa in the spermatheca of queens inseminated with semen from drones exposed to Fipronil could lead to a greater proportion of unfertilized eggs laid, resulting in a deficit of worker brood. The probability of having unfertilized eggs, from the exclusive release of dead spermatozoa, is especially important because the queen is economical in terms of sperm use^64^. If the queen releases only live spermatozoa, defects in brood production would potentially result from altered spermatozoa functional and structural integrity, particularly that of membranes or DNA, which have been shown to be affected by oxidative stress in rats after exposure to Fipronil^38^. DNA damage has also been observed in insect spermatozoa, especially in Drosophila, subsequent to a redox homeostasis impairment induced by an environmental stressor^65^. Therefore, it is legitimate to consider that similar effects could occur in honey bee spermatozoa, which exhibited a high reducing potential subsequent to drone exposure to Fipronil; however, further research is required to support this hypothesis. If such a hypothesis is demonstrated in the honey bee, DNA damage could be induced by Fipronil or by its metabolites, leading to DNA modifications that could result in mutations or disrupted epigenetic processes^66^. This assumption is supported by the ability of Fipronil to impact mitochondria^31^,^47^,^48^, which are critical for epigenetic regulation and the response to DNA damage^67^. Following exposure to environmental pollutants, including pesticides, transgenerational effects, such as a decrease in fertility and/or some developmental delays, can affect offspring health over several generations, as observed with unexposed F1 individuals in other insects species^11^,^12^,^68^. Hence, we hypothesize that similar detrimental effects may occur in honey bees. However, more investigations are needed on vertical intercolony transmission, which could also occur via queens carrying DNA modifications (generation ≥ F1) and drones issued from these queens. If this assumption was demonstrated for drones, which are haploid with the genetic material of queens because they develop from unfertilized eggs, the transmission of altered genetic material could occur only from F2. Consequently, the indirect effects of pesticides, such as those observed in this study in queens at an individual level, could be extended to the colonial level, even for the colonies and their lines that are never exposed to the original stressor. This mode of pesticide effect transmission could adversely impact the species and explain the decline of honeybees, at least in part. This situation may be worsened by the exposure of honey bee reproducers to a myriad of pollutants, including other pesticides^69^, that can act alone or in synergy^70^, or interact with infectious agents and parasites^71^,^72^.

Our findings do not enable the exclusion of effects on a queen’s offspring, but they do strongly suggest that poor drone quality leading to poorly-mated queens could be a mechanistic explanation for queen failure. To confirm this assumption, the next step would be to monitor colonies in field conditions after the reintroduction of artificially inseminated queens. An interesting further step would be to evaluate the capability of exposed drones of transmitting their semen in a natural mating context. A strong competition between drones occurs in this context, and the success of drone mating could be studied. Thus, if exposed drones are unable to mate, no altered semen will be present in the spermatheca. However, fertility may be impaired without altered drone vitality, as observed for humans, who exhibit a constant decay in fertility for more than 50 years^5^. Hence, drones with reduced fertility could have equivalent chances of mating as unexposed drones. Thus, if they succeed, the poor semen provided by the exposed drones could be diluted in the spermatheca by healthy semen because honey bee queens mate with up to 20 drones^55^,^60^ and have an excess of spermatozoa. Consequently, it cannot be excluded that such a mating would result in non-observable effects on the offspring. However, the chances of observing healthy males exhibiting no decreases in fertility might be relatively low. In natural conditions, colonies and consequently drones are exposed to numerous environmental stressors due to the ubiquity of biotic stressors^13^ and chemicals in hives^73^,^74^. Therefore, it seems more likely that a generalized decrease in drone fertility would occur and result in an impoverishment of semen stored by queens, as demonstrated with Fipronil, leading to queen failure.

For honey bees, the existence of such fertility impairments, in addition to sublethal effects, at very low levels of exposure, raises the question on the environmental risks generated by the use of systemic pesticides, such as phenylpyrazoles and neonicotinoids. Thus, reproductive disorders induced by agrochemicals on honey bees must be also taken into consideration in the assessment of pesticide risks.

In the context of declining pollinator populations^75^, it would be surprising for reproductive disorders to be restricted to honey bees without affecting other pollinating insects. This reduced fertility could lead to the loss of many species of pollinating insects, which in turn threatens plant reproduction and, consequently, biodiversity. Thus, the disruption of reproductive function by environmental stressors, such as agrochemical pollutants and biological agents, can be regarded as a catalyst of the species extinction phenomenon.

## Methods

Experiments were performed in Avignon (France) between early May and late July during 3 consecutive years (2012, 2013 and 2014). To perform the experiments, drones, queens, workers and brood combs were obtained from managed honey bee colonies (*Apis mellifera* L). These colonies were treated each year in September with Amitraz to control *Varroa* mite pressure. To evaluate the reproductive toxicity of Fipronil on drones, 4 experiments were conducted (1 in 2012, 2 in 2013 and 1 in 2014). The impact of these exposures on queen reproductive potential was only investigated in summer 2014.

### Drone rearing, exposure to Fipronil and food consumption

Twenty-five days before the experiments began, queens of 10 colonies were caged 2 days on drone combs to control drone production (1 comb/colony). One day before drone emergence, the combs were recovered and introduced into the breeder colony with workers. After emergence, a homogeneous set of 300 drones from all brood frames was locked in uniform, queenless colonies that are known to take better care of drones^76^. These newly prepared colonies were composed of 5000 workers, one brood frame, and four empty frames with no food storage to prevent Fipronil dilution in food, as described by Ben Adbelkader *et al*.^77^. These colonies were grown under a tunnel covered with an insect-proof net (two colonies per compartment). For 20 days, in each tunnel compartment, colonies were supplied daily by foragers that could harvest sugar syrup (50% w/v) *ad libitum* from a feeder from 8:30 a.m. to 11:30 a.m.; the syrup was replaced with crushed pollen and water for the remainder of the day^77^. Control and exposed hives were provided sugar syrup (0.1% DMSO) only or sugar syrup contaminated with Fipronil at 0.1 μg.L^−1^ (0.1% DMSO), respectively. Stock solutions of Fipronil (1 μg.L^−1^) were used each year and prepared in 1% DMSO for dilution in sugar syrup to a final concentration of 50% sucrose, 0.1% DMSO and 0.1 μg.L^−1^ Fipronil, as confirmed by GC–MS/MS chemical analysis, with a mean value ± SD of 1.08 ± 0.11 μg.L^−1^ (n = 3).

The amounts of syrup and pollen foraged were measured daily in each compartment, and the evaporation rate was not measured as it was assumed to be identical in all compartments. Thus, during their sexual maturity, drones were chronically fed with a food gathered to the hive by foragers, as in natural conditions. A chemical analysis of the Fipronil and its metabolites in honey stored in exposed hives was performed using GC–MS/MS, and no substances were detected (LOD = 0.2 μg/kg). The set of experiments contained 22 control and 22 exposed colonies (4 and 4 in 2012, 6 and 6 for the first experiment in 2013, 4 and 4 for the second experiment in 2013, and 8 and 8 in 2014). At the end of the experiments, i.e., 20 days, the drones were captured for semen collection.

### Drone survival rate, maturity rate and semen collection

Drones were exposed to Fipronil from emergence. After 20 days of exposure, coinciding with their sexual maturity, drones from each colony were caught, and the survival rate was recorded. Semen was subsequently collected immediately with a glass capillary tube connected to a syringe filled with Kiev solution (36 g/L trisodium citrate, 3.6 g/L sodium bicarbonate, 0.6 g/L potassium chloride, 5 g/L glucose, 3 g/L sulfanilamide, pH 8.5, osmotic pressure 460 mOs/mL)^77^.

For each hive, semen from different drones was pooled in the same capillary tube. During semen collection, the maturity rate of drones from each colony was assessed by the ability to provide sperm after stimulation. The average semen volume per drone was also determined, considering the number of drones providing semen and the total volume collected measured in calibrated glass capillary tubes. The spermatozoa concentration, mortality rate and metabolic activity (as assessed by measuring the reducing potential and adenosine triphosphate (ATP) content) in fresh semen were assessed. In 2014, semen samples from exposed and control drones were used to inseminate two groups of sister queens.

### Characterization of semen quality

#### 1) Spermatozoa concentration

Fresh semen was diluted (1:1500) in Kiev solution before the spermatozoa were counted under a phase-contrast microscope using a cell counter (Neubaeur improved/Petroff). For each sample, 5 counting iterations were performed to determine an average value.

#### 2) Mortality rate of spermatozoa

The spermatozoa mortality rate was determined using a conventional dead cell stain, propidium iodide, from the LIVE/DEAD Sperm Viability Kit (Molecular Probes L-7011) as recommended by the supplier. Briefly, samples of diluted semen were analyzed by fluorimetry (FI λ_ex_ = 535 nm, λ_em_ = 617 nm) in triplicate in a 96-well black microplate in a TECAN infinite F500 plate reader. Each well contained 100 μL of diluted semen in Kiev solution containing 1 × 10^7^ spermatozoa and 60 μM propidium iodide. Before reading the fluorescence, the microplate was incubated in the dark for 10 min at 34 °C. Spermatozoa mortality was determined as a percentage from the fluorescence intensity using a standard range up to 1 × 10^7^ dead spermatozoa obtained by successive freezing and thawing.

#### 3) Reducing potential in sperm

The reducing potential was determined using a Prestoblue kit (Invitrogen), in which resazurin is reduced by cell metabolic activity. Semen was analyzed in triplicate using a 96-white-well microplate and a TECAN infinite F500 plate reader. Each well contained 90 μL of diluted semen in Kiev solution containing 1 × 10^7^ spermatozoa and 10 μL of Prestoblue. Before reading the absorbance at 570 nm, the microplate was incubated in the dark for 10 min.

#### 4) ATP content in sperm

ATP content was determined using an ATPlite kit from PerkinElmer in the same wells used to assess the reducing potential, as recommended by the manufacturer and described by Ben Abdelkader *et al*.^77^.

### Queen rearing and instrumental insemination

In 2014, in parallel with the exposure experiment, queens were reared for instrumental insemination (at 8 days old) one week after drone semen collection. To obtain sister queens, young larvae (less than 24 h) from a single brood frame were grafted in artificial queen cell cups fixed to a frame and were subsequently introduced in a strong breeder colony. Two days before queen emergence, the queen cells and wax were fixed on top of Type Pain cages. Thirty newborn bees were also introduced in each cage to assist and feed the queens. Sucrose syrup (50% w/v), crushed pollen and water were given *ad libitum* to the bees. Cages were incubated at 34 °C and 60% RH until the queens reached sexual maturity for insemination with semen from capillary tubes previously stored in the dark at 21 °C, which is consistent with beekeeping practices. However, in another experiment, we verified that a storage period of 2 weeks in these experimental conditions did not alter sperm viability. This result is in accordance with those of previous studies^78^,^79^. In addition, the viability of spermatozoa was assessed one day prior to insemination. Before insemination, each queen received two 10-min CO_2_ treatments to simulate mating flight for better sperm acceptance and to contribute to the initiation of oviposition^76^. The first CO_2_ treatment was performed the day before insemination, and the second was performed on the day of insemination. The queens (40 per modality) were instrumentally inseminated with 8 μL of semen from control or exposed drones, corresponding to about 9.5 drones per queen. Newly inseminated queens were then placed in their respective cages with 30 new emerging bees and housed in the incubator for up to 2 weeks before analysis. After this period, the queens began to lay eggs regardless of modality.

### Characterization of spermatozoa stored in spermatheca

Fourteen days after insemination, of the 40 queens inseminated by each modality, 28 control queens inseminated with semen of unexposed drones and 30 queens inseminated with semen of exposed drones were still alive and were analyzed. Most deaths occurred a few days after insemination and may be attributable to injuries inflicted during the procedure. Queens were individually weighed and dissected in phosphate-buffered saline solution (PBS) to remove the spermatheca. After removal, the spermatheca was crushed in 500 μL of PBS to release spermatozoa and obtain a sperm suspension. The number of spermatozoa stored in the spermatheca and their mortality rate were determined.

#### 1) Number of spermatozoa

The spermatozoa suspension was diluted (1:3) in Kiev buffer to count the spermatozoa under a phase-contrast microscope using a cell counter (Neubaeur improved/Petroff). For each spermatheca, 5 counting iterations were performed to determine an average value.

#### 2) Spermatozoa mortality rate

The spermatozoa mortality rate was determined using a Vita-Eosin kit (RAL Diagnostics) adapted for honey bee sperm. Briefly, 10 μL of spermatozoa suspension was incubated with 60 μL of eosin stain for 5 min at ambient temperature, followed by the addition of 20 μL of nigrosine stain. Two smears were made on independent slides and observed under a phase-contrast microscope. Dead spermatozoa, which were permeable to the eosin stain, were stained pink, whereas live spermatozoa remained white with purple-bordered contours due to nigrosine. From each smear, at least 200 spermatozoa were observed to determine the number and percentage of live and dead spermatozoa in each spermatheca.

#### 3) Living spermatozoa in spermatheca

The living spermatozoa in the spermatheca corresponded to the difference between the total number of spermatozoa stored in the spermatheca and the number of dead spermatozoa.

### Statistical analyses

The following analyses were performed using the lme4 package of R software^80^.

1) Statistical analyses of worker bee-foraging performance were performed in two phases: first, in by modeling foraging performance over time with a non-linear logistic function with two parameters (α = instantaneous growth rate of foraging, β = maximum quantity of cumulated forage); and second, by applying a generalized linear mixed model to the parameters α and β with a Gaussian distribution, setting identity as the link function and the year of the experiment as a random effect.

2) In the same way, a generalized linear mixed model was applied to analyze the effects of Fipronil on drone and fertility parameters.

3) The difference in queen weight between the control and treated subsets was estimated by t-test after confirming normality. A generalized linear mixed model (random distribution with identity as the link function and hive from which the sperm capillaries originated as a random effect) was used to evaluate the significance of spermatozoa stored in spermatheca and the relationships among drone semen characteristics and spermathecal content. No random effect was observed for the hive from which the sperm capillaries originated.

## Additional Information

**How to cite this article**: Kairo, G. *et al*. Drone exposure to the systemic insecticide Fipronil indirectly impairs queen reproductive potential. *Sci. Rep.* **6**, 31904; doi: 10.1038/srep31904 (2016).

## Figures and Tables

**Table 1:** Effects of chronic Fipronil exposure on drone life cycle traits.

	Ctrl (n = 22)	Fip (n = 22)	*p value*
	mean ± SD	mean ± SD	
Drone survival rate (%)	45.3 ± 22.8	39.8 ± 22.4	0.296
Drone maturity rate (%)	62 ± 9.6	64.3 ± 10.1	0.405
Semen volume (μL/drone)	0.83 ± 0.11	0.85 ± 0.11	0.602

At sexual maturity, which corresponded to the end of the exposure period, the hives were opened in order to capture 20-day-old cloistered drones and to collect semen. Drone survival and maturity as well as the average semen volume per drone were determined for each hive. The data represent the mean values ± standard deviations obtained from drone populations recovered from hives in 4 experiments monitored from 2012 to 2014 (n = 22). Statistical analyses were performed using a generalized linear mixed model with a random effect on the different experiments.
